# Vaginal high-grade sarcoma in pregnancy

**DOI:** 10.1016/j.gore.2021.100881

**Published:** 2021-10-15

**Authors:** P. Akametalu, J.M. Barcelon, O. Myint, N.A. Moatamed, B.Y. Karlan, M. Kamrava, J.G. Cohen

**Affiliations:** a University of California Los Angeles, Division of Gynecologic Oncology, Los Angeles, CA, USA; b Olive View Medical Center, Department of Obstetrics and Gynecology, Sylmar, CA, USA; c University of California Los Angeles, Department of Pathology, Los Angeles, CA, USA; d Cedars-Sinai Medical Center, Department of Radiation Oncology, Los Angeles, CA, USA

**Keywords:** Vaginal sarcoma, Pregnancy, Radiation

## Abstract

**Background:**

Vaginal cancer is a rare malignancy making up 1–2% of all female genital tract cancers. Among vaginal cancers, sarcomas constitute 2% of malignant vaginal lesions, with leiomyosarcomas being the most common type of sarcoma. There is a paucity of data to guide treatment of vaginal sarcomas. This case report details a patient diagnosed with a gynecologic sarcoma during pregnancy who is subsequently treated for residual vaginal disease in the postpartum period with local resection and adjuvant vaginal brachytherapy.

**Case:**

A 31-year-old gravida 4 para 0 who presented at 22-weeks gestation with vaginal bleeding to an outside hospital and expelled a mass 11 cm in diameter from the vagina during her admission. Findings were consistent with a high grade gynecologic sarcoma. She underwent planned cesarean section at 36 weeks gestational age with uterine pathology showing no sarcoma. At her 3 month postpartum visit she was found to have a 1 cm posterior vaginal wall lesion which was resected and consistent with vaginal sarcoma. She underwent adjuvant brachytherapy.

**Conclusion:**

This case demonstrates the challenges with obtaining a correct pathological diagnosis for pregnant patients with vaginal sarcoma during pregnancy. Surgical resection with negative margins remains an important treatment component. Given the low incidence of disease occurrence in pregnancy and rare number of cases reported in literature, further elucidation of timing of delivery and adjuvant treatment is warranted.

## Highlights

• Vaginal sarcoma is rare in pregnancy.
• Surgical excision is the mainstay of treatment.
• Adjuvant radiation may improve local control in the management of vaginal sarcoma.

## 1 Introduction

Vaginal cancer is a rare malignancy making up 1–2% of all female genital tract cancers (Adams and Cuello, 2018). 1,252 women (0.6 per 100,000 women) were diagnosed with vaginal cancer in the United States in 2018 (U.S. Cancer Statistics Working Group, 2021). Sarcomas constitute 2% of malignant vaginal lesions, with leiomyosarcomas being the most common type of sarcoma (Matsuo et al., 2009). While we have limited data on vaginal sarcoma, clinical risk factors for uterine sarcoma include ethnicity, increased age, and exposure to Tamoxifen or pelvic radiation (Wickerham et al., 2002). Most uterine sarcomas are diagnosed in the sixth decade of life, with black women having a two-fold increased risk of leiomyosarcoma compared to white women (Chan et al., 2008). Genital sarcomas in pregnancy are extremely rare. Up to 0.1% of women are affected by cancer during pregnancy (Han et al., 2014). A review by Matsuo et al. covering the period 1955–2007 found a total of 5 cases of female vaginal sarcomas diagnosed during pregnancy (Han et al., 2014, Schwartz et al., 1963, Roddick and Honig, 1965, Mhaskar et al., 1989, Singhal et al., 1990, Behzatoğlu et al., 2003). Since then, only 2 additional cases of a patient with vaginal sarcoma during pregnancy have been reported in the literature (Church et al., 2010, Bassa et al., 2015). Here we report a case of a high-grade vaginal sarcoma diagnosed during pregnancy. Informed consent has been obtained from the patient for presentation of this case. This case report details a patient diagnosed with gynecologic sarcoma during pregnancy who is subsequently treated for residual vaginal disease in the postpartum period with local resection and adjuvant vaginal brachytherapy

## 2 Case report

A 31-year-old gravida 4 para 0 African American woman at 22-weeks gestation presented with vaginal bleeding to an outside hospital. Her obstetric history was significant for 2 therapeutic abortions and 1 spontaneous abortion. During prenatal care, the fetus was noted to have a unilateral dysplastic kidney. The patient’s family history was significant for two family members with cervical cancer and two family members with endometrial cancer. She was placed on bedrest with inpatient admission. 48 h after admission she developed pelvic pain and uterine contractions. She expelled a mass vaginally measuring 11 × 9 × 5 cm with no fetal contents. The pathology from the outside facility showed a showed a highly cellular tumor composed of spindle-shaped cells and bizarre multinucleated giant cells with focal myxomatous change with mitotic count is greater than 50 per 10 high power field (Positive for CD10, SMA, ER, PR, EMA) with the differential diagnosis including endometrial stromal sarcoma and undifferentiated uterine sarcoma. The patient underwent examination under anesthesia, demonstrating a 3 × 3 cm defect of the posterior vaginal wall with active bleeding, which was sutured for hemostasis. The cervix was long, closed, and high with no evidence of bleeding and the fetus was intact with normal heart tones. She was discharged in stable condition.

At 27-weeks, the patient was referred to our institution for maternal-fetal medicine, and gynecology oncology consultation. On initial evaluation, she reported no prior abnormal gynecology history and no history of infertility issues. She denied dyspareunia and bulk symptoms prior to or during pregnancy, and her periods before pregnancy were normal. A pap smear at 27-weeks gestation was normal and negative for human papilloma virus. Her intake physical exam at our institution revealed a normal pelvic exam with no evidence of residual vaginal mass, and an ultrasound revealed no intrauterine myomas.

Given the differential diagnosis included an endometrial stromal sarcoma, an undifferentiated uterine sarcoma, and a primary vaginal sarcoma, the patient underwent magnetic resonance imaging (MRI) without contrast of the chest, abdomen, and pelvis at 27 weeks. MRI findings were notable for a peri-centimeter cyst in the cervix consistent with a Nabothian cyst, a multiloculated cystic structure in the fetal abdomen consistent with dysplastic kidney, and an anterior placenta. There was no evidence of metastatic disease.

Due to the inability to ascertain whether the cancer had originated in the uterus, cervix or vagina, the patient desired definitive management for possible uterine sarcoma. In consultation with maternal fetal medicine and gynecology oncology specialists, the decision was made to proceed with a cesarean hysterectomy at 36 weeks. Betamethasone 12 mg intramuscularly was given for two doses 24 h apart with the first dose at 35 weeks and 6 days gestational age.

At 36 weeks and 1 day, she underwent planned exam under anesthesia of the vaginal canal with cesarean hysterectomy, bilateral salpingectomy, and peritoneal biopsies. She delivered a male infant weighing 2520 g with Apgars of 9 and 9. Operative findings were notable for normal appearing pelvic organs. There were no palpable or visibly enlarged pelvic/periaortic lymph nodes or evidence of metastatic disease throughout the peritoneal cavity. The neonate was admitted to the NICU post-delivery for prematurity. Neonatal US confirmed a right multi-cystic dysplastic kidney. He was transferred out of NICU on day 2 of life, and discharged home with his mother.

**Fig. 1:**
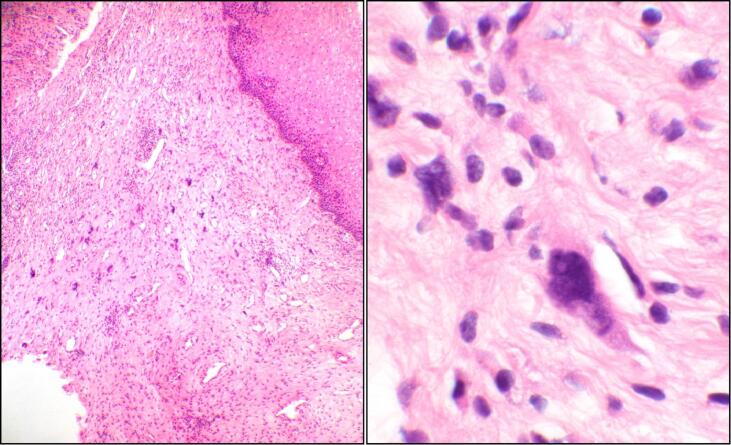
**Vaginal sarcoma excision:** Left panel is a low magnification of hematoxylin and eosin-stained section of the lesion showing pleomorphic spindle-shaped tumor cells infiltrating submucosa of the vaginal tissue (4× objective). Right panel is a high magnification of the same field further demonstrating the hyperchromatic nuclei with high nuclear to cytoplasmic ratio in detail (60× objective).

**Fig. 2:**
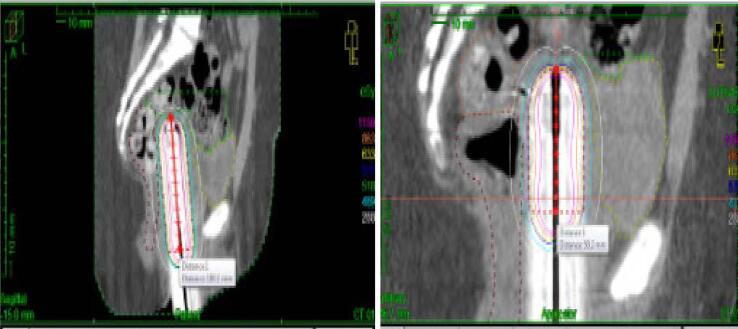
**Vaginal Brachytherapy Treatment Plan** The image on the left is a sagittal CT image with the initial plan treating the whole vagina followed by a boost field on the right to a smaller area covering the post-operative bed.

The final surgical pathology report was benign: no malignant or neoplastic cells were seen. The patient was seen at her 6-week postpartum visit and was doing well with a normal pelvic exam. At a follow-up surveillance visit 3 months postpartum, she was noted to have a 1 cm posterior vaginal wall lesion; biopsy results showed a high-grade sarcoma. The tumor was estrogen receptor and progesterone receptor positive. Computer tomography (CT) with contrast of the chest, abdomen, and pelvis showed no evidence of metastatic disease. The patient underwent an upper vaginectomy and proctoscopy. Operative findings were notable for 1.5 cm polypoid lesion in the posterior vagina wall 5 cm distal to the posterior vaginal apex. A 1 cm surgical margin was obtained circumferentially around this lesion where feasible, and surgical pathology showed high-grade sarcoma (Fig. 1) of the polyp lesion with negative surgical margins. The tissue removed from this excision showed residual sarcoma, morphologically similar to the original pathology from the outside hospital pathology. All other vaginal biopsies were also negative for malignancy. Marker seeds were placed in the proximal and distal margins of the areas where the sarcoma was excised. She was treated adjuvant high dose rate vaginal brachytherapy using a multi-channel vaginal cylinder. She received a biologically equivalent 2 Gy dose (EQD2) of 45 Gy to the whole length of the vagina and 60 Gy to the post-operative bed (Fig. 2). A CT scan 36 months after surgery continue to show no evidence of disease. She remains disease free 58 months after completion of vaginal brachytherapy.

## 3 Discussion

Primary cancers of the vagina are rare and encompass 1% of all malignant neoplasms of the female reproductive tract with the majority made of squamous cell carcinomas (Bassa et al., 2015). Sarcomas of the vagina account for only 2% of vaginal malignancies and are exceedingly rare to occur during pregnancy (Bassa et al., 2015). As a result, treatment of vaginal sarcoma is not well defined and management recommendations are often extrapolated from other gynecologic and soft tissue.

Presentation of vaginal sarcoma at the time of pregnancy is often confounded by the possible concern for a uterine fundus or cervical sarcoma as the source of malignancy. In this case, the mass which was expulsed spontaneously from the vagina at 22 weeks gestational age was a gynecologic sarcoma of unclear origin. In retrospect, the unexpected vaginal lesion identified 3 months postpartum likely represented the origin of the tumor which had been oversewn in the operating room at the time of initial presentation prior to transfer from the outside hospital. Based on staging for vaginal cancer, she had FIGO stage I disease with the sarcoma confined to the vagina. While an exam of the vaginal canal was performed at the time of cesarean hysterectomy, the area of recurrence may have been microscopic or undetectable if oversewn at the outside hospital. A thorough examination of the vaginal canal is important during the initial evaluation and subsequent surveillance. Local excision of this vaginal lesion identified postpartum followed by targeted treatment with high dose rate vaginal brachytherapy resulted in a durable treatment response of 57 months.

In conjunction with a multidisciplinary team of gynecologic oncology and maternal fetal medicine, a delivery date of 36 weeks was chosen to reduce the risk of fetal lung immaturity given the concern for potential uterine sarcoma. The patient received 2 doses of betamethasone prior to delivery. Due to the inability to ascertain the primary origin of the gynecologic sarcoma, the patient desired definitive treatment and underwent a cesarean hysterectomy with bilateral salpingectomy. A lymph node dissection was not performed given there was no lymphadenopathy on imaging and the rate of lymphatic involvement in gynecologic sarcomas is very low (Nesrine et al., 2019). There is limited data to guide the role of ovarian preservation versus oophorectomy in premenopausal women with uterine and vaginal sarcomas. Retrospective data suggests in premenopausal women with stage I uterine leiomyosarcoma, ovarian preservation does not impact cancer survival (Nasioudis et al., 2017). The decision was made for ovarian preservation given the patient’s premenopausal age with no evidence of metastatic disease on imaging.

Genital sarcoma in pregnancy most commonly presents with abdominal pain, a growing mass, and or vaginal bleeding (Chan et al., 2008). When gynecologic sarcomas are identified in pregnancy, consideration should be given to the possibility of vaginal origin. In addition, the histologic subtype of sarcoma is important in delineation of treatment recommendations. Of the 7 reported cases of sarcoma in pregnancy, 4 were leiomyosarcomas, 2 were rhabdomyosarcomas, and 2 were classified as sarcoma botyroides (Wang et al., 2015, Schwartz et al., 1963, Roddick and Honig, 1965, Mhaskar et al., 1989, Singhal et al., 1990, Behzatoğlu et al., 2003, Church et al., 2010, Bassa et al., 2015). Treatment for rhabdomyosarcomas consistent of multi-agent chemotherapy and radiation. In contrast, there is conflicting data supporting the benefit for adjuvant radiation or chemotherapy with undifferentiated sarcoma, leiomyosarcoma, and endometrial stromal sarcoma.

In this clinical scenario, the pathology was most consistent with a high grade or undifferentiated sarcoma. Unlike uterine leiomyosarcoma, radiation may have a more likely role in the management of undifferentiated vaginal sarcomas. An exact negative margin measurement to minimize risk of recurrence is unknown, but obtaining a negative margin is important.

A review article of vaginal sarcomas in 1997–2012 in a single institution in China discussed 8 cases of primary vaginal sarcomas with the mainstay treatment being surgery (Wang et al., 2015). In this cohort, there was significant variation after surgical excision with both chemotherapy and radiation included. Consistent with other retrospective cohorts, the majority of patients had vaginal leiomyosarcoma, however other histologic subtypes identified include endometrial stroma sarcoma, undifferentiated sarcoma, and adenosarcoma. Tumor grade and stage are associated with prognosis (Wang et al., 2015).

Our patient had an isolated case of vaginal high-grade sarcoma with negative surgical margins. Radical surgery would have required a colostomy given the close proximity to the rectum. Adjuvant high dose rate vaginal brachytherapy minimized the morbidity that would have been required with radical surgery such as an abdominoperineal resection. In conclusion, this case demonstrates the challenges with obtaining a correct pathological diagnosis for pregnant patients with vaginal sarcoma during pregnancy. Surgical resection with negative margins remains an important treatment component. Given the low incidence of disease occurrence in pregnancy and rare number of cases reported in literature, further elucidation of timing of delivery and adjuvant treatment is warranted.

## Author contribution

Pamela Akametalu and Joshua Cohen made significant contributions to the conception and design as well as the drafting and revising of the article. Neda Moatamed performed the pathology evaluation and contributed to revision of the article. Jasminemay Barcelon, Ohmar Myint, Beth Karlan, and Mitch Kamrava contributed to data acquisition, analysis, as well revising of the article.

## Declaration of Competing Interest

The authors declare that they have no known competing financial interests or personal relationships that could have appeared to influence the work reported in this paper.
